# Opinion paper: A regional feed action plan – one-of-a-kind example from East Africa

**DOI:** 10.1017/S1751731120001056

**Published:** 2020-10

**Authors:** P. Opio, H. P. S. Makkar, M. Tibbo, S. Ahmed, A. Sebsibe, A. M. Osman, E. Olesambu, C. Ferrand, S. Munyua

**Affiliations:** 1 FAO Sub Regional Office for Eastern Africa, Food and Agriculture Organization of the United Nations, United Nations Complex in Nairobi, UN Avenue, Gigiri, Block P, PO Box 30470, 00100, GPO, Nairobi, Kenya; 2 Sustainable Bioeconomy, Lammaschgasse 54/7, A-1210, Vienna, Austria; 3 FAO Subregional Office for the Gulf Cooperation Council States and Yemen Ministry of Climate Change and Environment Building 4C/4 (P floor), Street No. 6, PO Box 62072, Abu Dhabi, UAE; 4 FAO, Viale delle Terme di Caracalla, 00153 Rome, Italy; 5 IGAD Center for Pastoral Areas and Livestock Development (ICPALD), PO Box 47824-00100 Nairobi, Kenya

## Introduction

This opinion paper puts forward a case for formulation of a regional animal feed action plan (**RAFAP**) and highlights its potential benefits.

## A case for a regional animal feed action plan

Animal feed and feeding impacts almost all services and operation of the livestock sector, and the sustainability of this sector hinges on how animal feed is produced and fed (Makkar, 2016). Most regions in the developing world have a good natural resource base, favorable conditions and reasonably good availability of ingredients for animal feeds; however, the lack of national animal feed policy, strategy and institutional framework to support the animal feed sector are the major constraints, hindering the feed sector growth. Livestock sector development strategies are operational in several countries, but they neglect the feed sector, resulting in poor impact in terms of increase in farmers’ income and overall sustainability of the livestock sector. Moreover, in some continents such as Africa, there is a large movement of animals, and natural resources including feed resources are shared among them. As a result, a transnational or a RAFAP and its implementation would enable efficient and sustainable use of such resources, including promotion of safe and quality feed-resource trade. In the feed sector, trade-offs could exist among environment, safety, quality, ethics including animal welfare and pricing dimensions in a region. Most feed-related issues are common to countries in a particular region and can be better addressed when acted in a concerted manner – *national action with a regional benefit*. The RAFAP could serve as one of the most prudent mechanisms to address common feed issues and challenges and to find solutions in a region.

In light of the above and to address other feed-related challenges, countries in East Africa considered it vital to develop an East African Animal Feed Action Plan (**EAAFAP**). This is the only action plan in the area of animal feeding, developed at a regional level (FAO and IGAD, 2019a). An overview of the plan is given in Table 1. To the best of our knowledge, most regions in the developing world, for example, Latin America and Caribbean, Asia or Africa have a common animal disease control plan but lack a RAFAP to address common animal feed issues and challenges across several nations in a region.

**Table 1:** An overview of the action plan

Priority areas	Expected outputs
1. Establish and strengthen animal feed data, information, reporting and communication systems	Regional feed resource requirements and availability assessed and documented
	Early warning and early action mechanism established
	Reporting and communication system developed
2. Develop sustainable animal feed supply chains	Feed assessments conducted by agro-ecological zone/production area
	Availability of and accessibility to feed (production, harvesting, conservation and storage) assessed
	Potential animal feeds identified and established for local feed production
	Animal feed business centers identified and operationalized
	Feed supply chains established, and emergency mechanism specified
	Emergency livestock feed supply coordinated with other interventions
3. Identify the status of rangelands and grazing areas and disseminate best practices for their management	Status of rangeland and grazing areas determined
	Evidence-based best practices on rangeland and grazing management disseminated and adopted
	Improve governance and management of rangelands
4. Strengthen an enabling environment for feed production	Policy, institutional and process frameworks and standards supported
	Capacity for feed production, processing and marketing strengthened

Duration of the plan: 5 years. It is the result of 12 months of consultative and participatory process building on experiences and lessons learnt by a wide spectrum of key stakeholders in public and private sectors, notably policy makers, academia, traders, pastoralist and farmers’ organizations, civil society, non-government organizations and development partners. It builds on the earlier consultative experience sharing workshop on feed by United States Agency for International Development, International Livestock Research Institute, IGAD and FAO in the region. Its implementation to be monitored and evaluated by regional animal feed platform set up by IGAD and its member states.

There could be several common challenges and solutions for the livestock sector in developing countries that geographically extend from Africa to Asia and Latin America. To address such issues in a focused manner and given that administrative regions (e.g. East Africa, West Africa, Latin America, South East Asia, among others) do coordinate implementation of interventions, a regional feed action plan could have an important role in sustainable development of the animal agriculture.

## The East Africa Animal Feed Action Plan and its benefits

Understanding livestock data, disaggregated by livestock production system and type of animals, and generation of feed requirements and feed inventory, as envisaged in the EAAFAP (Table 1), would be key in designing sustainable livestock production systems in East Africa. Improving data and information on the feed resources and animal feed supply chains is critical for countries to study and map strategic points to which feed investments can be targeted. This improved data would be a part of the *Big data* which the countries wish to build, following the spirit of the famous management quote*, if you cannot measure it you cannot manage it.* This is a pre-requisite for developing sustainable feed supply chains (Priority Area 2 of the Plan), both for lowlands and highlands in East Africa, respectively, hosting largely grazing and crop-livestock mixed systems. As an example, an assessment of availability and utilization of agroindustrial by-products in Ethiopia (FAO, 2019) has initiated discussions between the breweries and feed manufacturers to efficiently utilize the brewers grains as animal feed. This assessment also contributes to mitigation efforts of wastage of by-products and to diverting them to animal feed. Similarly, establishment of feed inventory and feed balance (Makkar *et al.*, 2019) has helped the Government of Ethiopia and other stakeholders including the private sector to: (a) identify zones of excess feed availability, (b) consider the use of compaction technologies to densify the feed to decrease the cost of transport and to supplement them during compaction to enhance feed quality and (c) assist establishing feed banks close to deficit and drought-prone areas, well before a drought strikes. Such efforts could also benefit other countries in the region facing similar challenges. These efforts of generating feed inventory and feed balance in African countries are in line with the inventory of animal feed availability and demand done by the EU countries (FAO, 2012).

Livestock feed and feeding systems are constrained by a host of interconnected factors, including and not limited to recurrent droughts and floods, state restrictions of livestock mobility, grassland degradation, overgrazing, land tenure and land use changes, resource use conflicts, encroachment of invasive plant species, soil infertility and inadequate pasture inputs and planting material. Seasonal feed and water shortages and inefficient use by pastoralist and agro-pastoralist communities are the major challenges affecting livestock productivity in Africa. These challenges are being addressed in the Plan through Priority Area 3 that would use models and remote sensing methodologies (Matere *et al.*, 2019) to identify the status of rangelands and grazing areas and disseminate best practices for their management. It also includes forecasting of forage and water availability in the rangelands, resulting in better preparedness in the dry areas by the governments and pastoralists in the region, as has been the case in Kenya (Matere *et al*, 2019). The biomass and water forecasting and assessment models have also been used in other countries under normal and emergency situations (FAO, 2012; Angerer *et al.*, 2014; Makkar *et al.*, 2020). Assessment of status of the rangelands is also a part of the *Big data*, complementing the Priority Area 1 of the Plan. The methodologies for assessing feed balance and forecasting of rangeland biomass are succinctly presented in our recent article and have been used in a number of countries (Makkar *et al.*, 2020). These efforts would help policy makers to formulate sound policies based on sound data, donors to allocate resources more rationally, feed industry to make efficient use of available resources to develop sustainable feed supply chain and researchers to develop appropriate feeding strategies. The conducive polices and participation of the private sector would strengthen an enabling environment for quality and safe feed production, making the feed sector more dynamic. To achieve large-scale impact of an innovation, informed policies and decisions together with knowledge of social and cultural behavior to adoption of the innovation are vital, thereby forming as integral pillars of the plan.

The plan provides opportunities to: (a) leverage the potential and opportunities provided by animal feed resources to stimulate development and income generation in rural communities by improving the efficiency and profitability of the animal feed sector; (b) enhance the participation of rural communities in the animal feed value chain; (c) facilitate community led small-scale feed enterprise and private sector-driven animal feed market development, within and outside East Africa, ensuring market access and competitive prices across countries; (d) guide governments to develop evidence-based enabling policies and regulatory frameworks on feed for enhanced trade between countries in East Africa and (e) exploit the production potential of rangelands and ensure sustainable natural resource use as a key ingredient in the development of the animal feed sector in East Africa.

## Implementation of the plan

Intergovernmental Authority on Development (**IGAD**) and Food and Agriculture Organization of the United Nations (**FAO**) expect that the EAAFAP will be used largely as a framework for countries to adapt to their own specific animal feed context and needs, with a broader objective to guide and support sustainable livestock-based livelihood development and resilience. Both organizations have shown commitment to achieving this plan through awareness creation at all levels, the promotion of technically feasible actions and advocating for countries to harmonize policies and legislative frameworks on feed to stimulate livestock sector growth in East Africa.

Implementation of the EAAFAP may face some challenges, notably financial, especially in South Sudan, Somalia and Eritrea where budgets are constrained due to ongoing political instability. In addition, the lack of human resources with the required level of skills and experience to implement the plan is acknowledged to be a constraint across member states. Intergovernmental Authority on Development and Food and Agriculture Organization of the United Nations plan to organize a series of awareness and joint resource mobilization efforts involving East African countries and ensure that national capacity is built to successfully implement the plan. In February 2020, both organizations conducted a workshop in Nairobi, Kenya in which representatives of all the IGAD countries participated and a National Animal Feed Security System was developed (Makkar *et al.*, 2020). The aim was that all countries in the region use harmonized protocols, enabling meaningful comparison of results produced by using the system. This was possible only through the EAAFAP.

Ethiopia and Kenya have initiated the process to generate sound animal feed data including the availability of grazing biomass and water in the rangelands for informed decision making (Makkar *et al.*, 2019 and 2020). The team members undertaking this work of generating sound data are from ministries, research organizations, universities, non-government organizations and international organizations. The role of researchers and academia in generating *missing numbers* is vital for successful completion of the Priority Areas 1 and 3 and in developing innovations for achieving other priority areas listed in the strategy. Additional efforts are underway to mobilize resources with IGAD to out-scale the ongoing feed inventory and feed balance work to all its member states and to initiate work on Priority Areas 2 and 4. Encouraged by the progress made so far, IGAD is finalizing the transhumance protocol to further guide its member states on how to facilitate peaceful and sustainable pastoral mobility in the region (FAO and IGAD, 2019b). Other issues on the card are to formulate regional hay standards, enabling promotion of quality and safe hay trade at a regional level which is extensive during droughts, and to harmonize feed quality and safety standards in the region. An example is the use of varying aflatoxin standards for animal feed in different African countries (Sirma *et al.*, 2018) that needs harmonization.

## Conclusion

In our opinion, the EAAFAP is a good example for other regions (in Africa and elsewhere) to consider formulating their respective feed plans particularly where feed resources are shared across communities and countries and where the livestock sector faces similar challenges. Learning from each other, translation of solutions and adapting policies from one country to another (South-South Cooperation) are faster and easier through a regional or a transnational plan. Same is the case for harmonization of the feed quality and safety regulations, which is essential for promotion of feed ingredient and feed trade among the countries in a region. Such plans are vital for enhancing development and resilience of the livestock sector in developing countries.

## Software and data repository resources

None of the data were deposited in an official repository.
